# Transfer of open and laparoscopic skills to robotic surgery: a systematic review

**DOI:** 10.1007/s11701-022-01492-9

**Published:** 2022-11-22

**Authors:** Baldev Chahal, Abdullatif Aydın, Mohammad S. Ali Amin, Kelly Ong, Azhar Khan, Muhammad Shamim Khan, Kamran Ahmed, Prokar Dasgupta

**Affiliations:** 1 MRC Centre for Transplantation, Guy’s Hospital, King’s College London, 5Th Floor Southwark Wing, London, SE1 9RT UK; 2 Department of Urology, King’s College Hospital NHS Foundation Trust, London, UK; 3 Oxford University Clinical Academic Graduate School, Oxford, UK; 4 Department of Urology, Princess Royal University Hospital, Orpington, UK; 5 Urology Centre, Guy’s and St, Thomas’ NHS Foundation Trust, London, UK

**Keywords:** Open surgery, Laparoscopic, Robotics, Simulation, Skill transfer

## Abstract

Due to its advantages over open surgery and conventional laparoscopy, uptake of robot-assisted surgery has rapidly increased. It is important to know whether the existing open or laparoscopic skills of robotic novices shorten the robotic surgery learning curve, potentially reducing the amount of training required. This systematic review aims to assess psychomotor skill transfer to the robot in clinical and simulated settings. PubMed, EMBASE, Cochrane Library and Scopus databases were systematically searched in accordance with PRISMA guidelines from inception to August 2021 alongside website searching and citation chaining. Article screening, data extraction and quality assessment were undertaken by two independent reviewers. Outcomes included simulator performance metrics or in the case of clinical studies, peri- and post-operative metrics. Twenty-nine studies met the eligibility criteria. All studies were judged to be at high or moderate overall risk of bias. Results were narratively synthesised due to heterogeneity in study designs and outcome measures. Two of the three studies assessing open surgical skill transfer found evidence of successful skill transfer while nine of twenty-seven studies evaluating laparoscopic skill transfer found no evidence. Skill transfer from both modalities is most apparent when advanced robotic tasks are performed in the initial phase of the learning curve but quality and methodological limitations of the existing literature prevent definitive conclusions. The impact of incorporating laparoscopic simulation into robotic training curricula and on the cost effectiveness of training should be investigated.

**Supplementary Information:**

The online version contains supplementary material available at 10.1007/s11701-022-01492-9.

## Introduction

Robotic surgery has experienced a rapid increase in uptake, with a four-fold increase in the number of robot-assisted operations performed over the last decade [1]. The continuing favourability of robot-assisted surgery (RAS) is due to the numerous advantages it possesses over open surgery and conventional laparoscopy such as faster learning curves, eradication of the fulcrum effect and the ability to mirror the movements of the wrist and hand [2]. Furthermore, when compared to open procedures, RAS operations have resulted in more favourable patient-centred outcomes such as significantly decreased blood loss, length of hospital stay, transfusion rates [3] and lower post-operative pain medication use [4].

Therefore, as experienced surgeons switch to robot-assisted operations to capitalise on its advantages, it is now a priority to address the training needs of current and future surgeons. It is clinically important to know how far existing skills transfer for safe robot-assisted operations on patients, and whether prior open or laparoscopic experience complicates or complements the robotic skill acquisition process. There are also implications for surgical trainees undergoing robotic training, based solely on the acquisition of robot-specific technical skills. Simulation forms the initial stage of robotic skills training due to its convenience, efficiency and provision of safe training posing no risk to patients [5]. Robotic simulators are more expensive [6] and less available than laparoscopic simulators [7, 8], so establishing whether skills learnt on laparoscopic simulators transfer to the extent that less time is needed on robotic simulators may optimise the use of such limited resources.

It might be expected that open surgical experience translates into enhanced robot-assisted performances as knowledge of the anatomy and approaches associated with operating in a particular region may aid in mastering the robotic form of the operation. Similarly, given that laparoscopic surgeons are well versed in fundamental minimally invasive techniques and accustomed to manoeuvring instruments through an indirect field of view, they might be well equipped with transferable skills to augment their robotic skills. This review aims to assess the transfer of open and laparoscopic psychomotor skills (gained through surgical simulation or operative experience) to the robot, evaluating their impact on the robotic learning curve. Successful crossover of skills across the various modalities would not only result in surgeons being able to operate across a range of settings but may optimise their training in terms of duration and cost effectiveness.

## Methods

### Design

A systematic review assessing the transfer of open and laparoscopic skills to robotic surgery was conducted in accordance with the Preferred Reporting Items for Systematic Reviews and Meta-Analyses (PRISMA) statement [9]. A review protocol was prospectively designed and registered on the International Prospective Register of Systematic Reviews (PROSPERO) database (CRD42021231235) [10].

### Eligibility criteria

Studies involving medical students, surgical trainees and consultant surgeons (expert surgeons) were included, with exclusion of any study involving non-medical participants. Eligible interventions included assessed performances of robotic tasks or procedures after undergoing laparoscopic training or having prior laparoscopic or open surgical experience. Comparators included control participants (no open or laparoscopic experience) or robotically trained participants. Outcomes included measures of task performance in operative or simulated settings, with studies not assessing the impact of skill transfer in statistical terms being excluded. Randomised controlled trials, non-randomised comparative studies, cohort studies and observational studies evaluating skill transfer were included. Review articles, editorials, letters to the editor and conference abstracts were excluded. A restriction on language was imposed, with only studies written in English being included.

### Search strategy

The PubMed, EMBASE, Cochrane Library and Scopus databases were systematically searched from their inception to August 2021. A combination of free-text terms and medical subject headings (MeSH) was used in the searches. For PubMed and the Cochrane Library, the search strategy ‘(laparoscop* OR open OR Laparoscopy[mesh] OR Minimally Invasive Surgical Procedures[mesh]) AND (robot* OR Robotic Surgical Procedures[mesh]) AND (transfer*)’ was used. For Embase, ‘((laparoscop* OR open) AND robot* AND transfer*)’ was used, while ‘((laparoscop* OR open) AND surg* AND robot* AND transfer*)’ was employed for Scopus. The Google Scholar search engine was employed in combination with website searching and citation chaining to find relevant grey literature.

### Study selection and screening

Initial screening of article titles and abstracts was performed by two independent reviewers (BC and MSAA). Duplicates were removed and the full text of articles which passed this initial screening process was then assessed for eligibility against the inclusion criteria. Disagreements over inclusion or exclusion were referred to a third reviewer (AA).

### Data extraction and risk of bias assessment

Data extraction was performed by two independent reviewers using a pre-defined, standardised form. Any disagreements were resolved by a third reviewer. Data extracted included study characteristics such as author, publication date, study type and study population, as well as study outcome measures and results. All included studies were assessed for risk of bias by two independent reviewers using the Cochrane risk-of-bias tool for randomised trials (RoB 2) [11] and the Risk Of Bias In Non-Randomised Studies of Interventions (ROBINS-I) tool [12]. Disagreement was resolved by referral to a third reviewer.

### Data synthesis

It was not possible to undertake a meta-analysis due to heterogeneity in study design and outcome measures. Thus, results were narratively synthesised in accordance with PRISMA [9] and Synthesis without meta-analysis (SWiM) [13] guidelines.

## Results

**Fig. 1:**
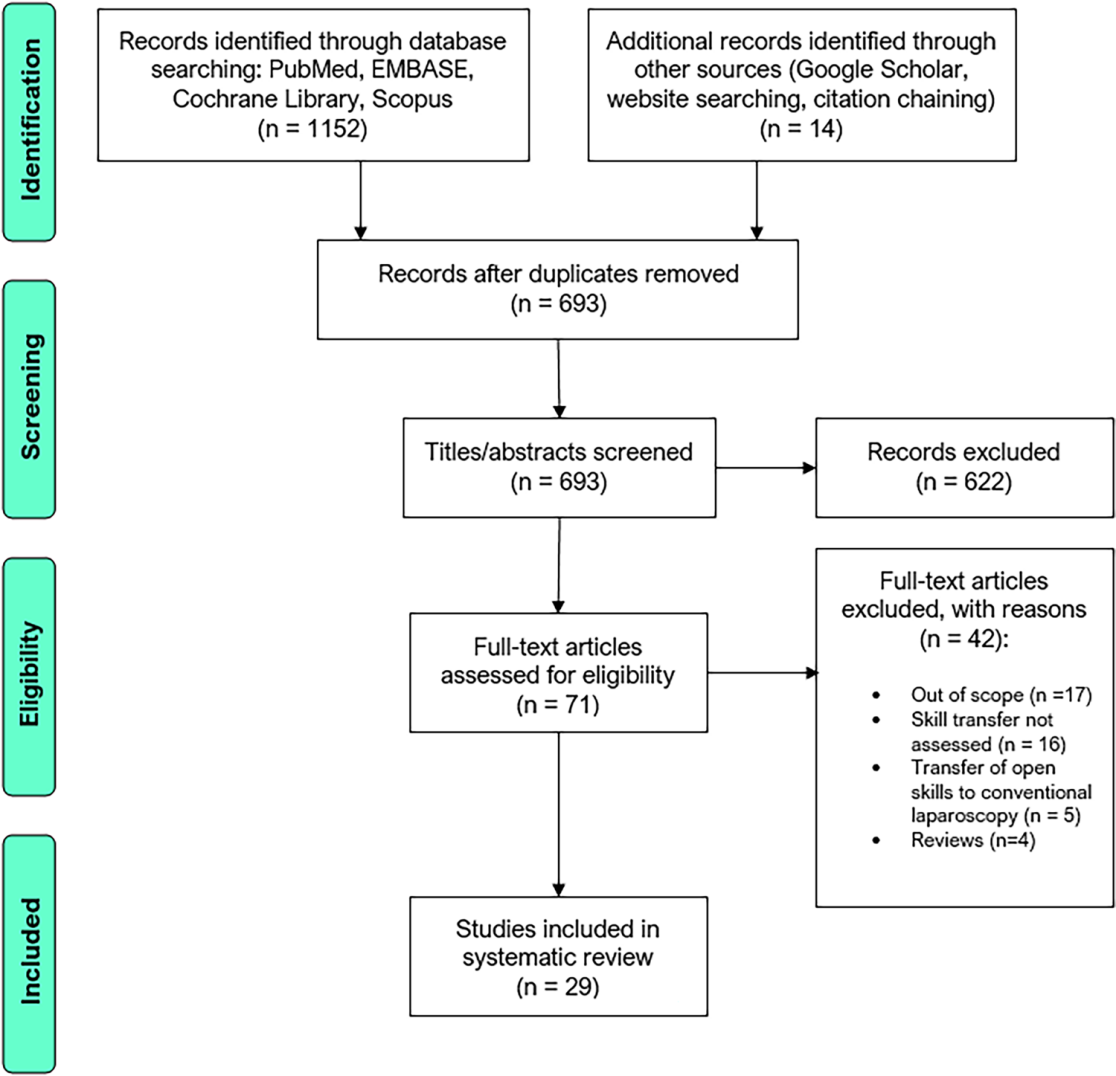
PRISMA flow diagram

One thousand one hundred and fifty-two records were identified through database searching and fourteen additional records were identified through a search of the grey literature and citations. After deduplication, 693 studies were eligible for title and abstract screening with 622 studies being excluded, leaving 71 studies for full-text screening (Fig. 1). Forty-two studies were then excluded after full-text screening. Thus, 29 studies were included for narrative synthesis.

### Characteristics of included studies

The characteristics of the included studies are displayed in Tables 1 and 2. Of the studies included were 9 randomised controlled trials [20, 21, 23, 24, 27, 32, 33, 38, 42] (RCTs), 15 prospective cohort studies [15, 16, 22, 25, 26, 28–31, 34–37, 39, 41], 2 non-randomised controlled trials [18, 40], 1 retrospective cohort study [17], 1 uncontrolled before-and-after study [19] and 1 prospective single-surgeon study [14]. The publication dates of the included studies ranged from 2003 to 2020. All studies had a small sample size ranging from the results of 1 surgeon [14] to 21 participants [30]. Study participants included medical students, surgical trainees of all levels of training and expert surgeons. Robotic systems used in studies included the da Vinci Surgical System (Intuitive Surgical, Sunnyvale, CA), the Mimic dV-Trainer (Mimic Technologies Inc., Seattle, Washington), the RoSS surgical simulator (Simulated Surgical Systems, Williamsville, New York) and the Senhance Surgical System (TransEnterix Inc., Morrisville, NC).

**Table 1:** Characteristics of included studies assessing skills transfer in clinical settings

Study author, year	Skill transfer assessed	Specialty, country	Participants	Procedure(s)	Comparison group(s)
Ahlering et al. [14], 2003	Open → R	Urology, USA	1 Urological oncologist (open exp. only)	45 robot-assisted lap. radical prostatectomy	None
Eddib et al. [15], 2013	Lap.→ R	Gynaecology, USA	64 cases performed by advanced lap. surgeons (lap. hysterectomy exp.)	Robot-assisted lap. hysterectomy	72 cases performed by intermediate lap. surgeons (no lap. hysterectomy exp.)
Kilic et al. [16], 2012	Lap. → R	Gynaecology, USA	7 Gynaecology residents with ≥ 3 lap. experiences	Vaginal cuff closure with either: robotic suturing with intracorporeal knot tying Lap. suturing with extracorporeal knot tying	6 Gynaecology residents with ≤ 2 lap. experiences
IK. Kim et al. [17], 2014	Lap. → R	Colorectal surgery, South Korea	First 100 consecutive cases performed by 1 surgeon (exp. of > 300 lap. colorectal operations)	Robot-assisted low anterior resection for rectal cancer	First 100 consecutive cases performed by 1 surgeon (exp. of < 30 lap. colorectal operations)

*exp*. experience, *lap*. laparoscopic, *R*. robotic

**Table 2:** Characteristics of included studies assessing skill transfer in simulated settings

Study author, year	Skill transfer	Participants	Task sequence	Comparison group(s)
Anderberg et al. [18], 2010	Lap. → R	10 medical students (SN) performing tasks with conv. lap. first then on the da Vinci robot	Repeated 4 times on each platform: Grab the Needle Continuous Suturing Tie a knot	10 medical students (SN) performing tasks with the robot first then with conv. lap
Angell et al. [19], 2013	Lap. → R	14 medical students (SN)	Spiral cutting exercise using da Vinci robot 1 month lap. training (no robotic exposure) Repeat spiral cutting exercise on da Vinci robot and on lap. box trainer	None
Ashley et al. [20], 2019	Lap. → R	15 medical students (SN) trained on a lap. simulator and assessed on robotic simulator (RoSS)	Balloon Grasp and Ball Drop tasks on RoSS, and Peg Transfer and Ball Drop tasks on lap. simulator Participants randomised to either lap. simulator or RoSS and perform Ball Drop 10 times Perform Ball Drop with the unpractised modality	16 medical students (SN) trained on RoSS and assessed on lap. simulator
Blavier et al. [21], 2007	Lap. → R	40 medical students (SN) performing tasks with conv. lap. before switching to the da Vinci robot, or vice versa	Needle Guidance task	Medical students performing task with the robot first, then with conv. lap
Cumpanas et al. [22], 2017	Open → R	10 trainee surgeons (> 2 years of open surgical exp. only)	3 repetitions on da Vinci Skills Simulator of: Peg Board Level 1 (easy) Energy Dissection Level 2 (intermediate) Suture Sponge Level 3 (difficult)	15 final year medical students (SN)
Davila et al. [23], 2017	Lap. → R	9 surgical trainees (RN with limited lap. exp.) trained on lap. simulator then assessed on the da Vinci robot	3 consecutive attempts each of Intracorporeal Knot Tying and Peg Transfer tasks performed on the robot Participants undergo 4 1-h training sessions in their assigned training modality Robotic skills then reassessed with the same two tasks	88888surgical trainees receiving no training 10 surgical trainees trained on da Vinci Skills Simulator
Feifer et al. [24], 2010	Lap. → R	5 medical students (SN) undergo ProMIS (Haptica, Ireland) practice 5 medical students (SN) undergo LapSim (Surgical Science, Sweden, AB) practice 5 medical students (SN) undergo ProMIS and LapSim training	Baseline evaluation on da Vinci robot with Peg Transfer, Pattern Cutting, Intracorporeal Suturing and Cannulation Participants undergo practice on assigned platform Reassessed on da Vinci robot	5 medical students (SN) undergo no lap. training
Finnerty et al. [25], 2016	Lap. → R	28 surgical residents (RN with varying lap. experience ranging from 1 to 750 cases logged)	1 trial on da Vinci robot of: Match Board Energy Dissection Suture Sponge	8 medical students (SN)
Hagen et al. [26], 2009	Lap. → R	16 lap. trained fellows or senior surgeons	10 repetitions on da Vinci robot of: Rubber Ring Placement Suturing with Knot Tying Needle Guidance	18 medical students or residents (LN)
Hassan et al. [27], 2015	Lap. → R	15 medical students and junior residents (SN) performing tasks with conv. lap. first then on da Vinci robot	5 repetitions on each platform of: Pick and Place Thread the Ring	15 medical students and junior residents (SN) performing tasks with R. lap. first then with conv. lap
Jayaraman et al. [28], 2010	Lap. → R	Lap. exp. surgeon (RN)	Each participant performed 10 lap. and 10 robotic choledochojejunostomies on ex vivo model consisting of porcine livers with contiguous intestines	Surgeon with R. and lap. exp Surgeon with only basic lap. exp
Kim et al. [29], 2014	Lap. → R	10 lap. exp. surgeons (RN)	Bead Transfer, Ring Insertion onto Cone, and Suturing with Knot Tying tasks performed on both lap. and da Vinci robotic platforms Tasks repeated after an 8-week interval	10 LN medical students
Kowalewski et al. [30], 2018	Open → R Lap. → R	25 surgeons (varying levels of open and lap. exp.)	1 repetition each on da Vinci robot of: Peg Board Pick and Place Thread the Rings Suture Sponge	37 medical students (SN)
McVey et al. [31], 2016	Lap. → R	32 trainees with varying levels of lap. exp	Participants undergo 4 week robotic surgery course Assessed pre- and post-course on R. and lap. platforms: Peg Transfer Intracorporeal Suturing and Knot Tying	Comparisons performed in analysis based on baseline lap. skill score
Moncayo et al. [32], 2019	Lap. → R	6 medical students (SN) performing tasks on the lap. platform first then on the R. platform	Repeated 5 times on each platform: Thread the Ring Transfer the Plot	6 medical students (SN) performing tasks on the R. platform first then on the lap. platform
Obek et al. [33], 2005	Lap. → R	10 medical students (SN) assessed with robotics first, undergo conv. lap. training then reassessed with robotics	Intracorporeal Knot Tying performed on assigned pre-training platform Knot Tying practiced with assigned training method Knot Tying reassessed on pre-training platform	10 medical students (SN) assessed with conv. lap. first, undergo R. training then reassessed with conv. lap
Panait et al. [34], 2014	Lap. → R	14 lap. exp. surgery residents (RN)	Tasks performed on lap. platform first and then on R. platform (after a 24 h interval): Peg Transfer (easiest task) Circle Cutting (intermediate) Intracorporeal Suturing (hardest task)	14 medical students with minimal lap. exp. and no R. exp
Passerotti et al. [35], 2015	Lap. → R	12 lap. exp. surgeons (RN)	Performed in 5 consecutive, weekly training sessions on both the lap. and robotic platforms: Peg Transfer Precision Cutting Suturing with Intracorporeal Knot Tying	31 SN
Pimentel et al. [36], 2018	Lap. → R	20 lap. exp. surgeons (RN)	4 repetitions on da Vinci-Trainer of the following sequence: Peg Board 2 Ring and Rail 1 Suture Sponge 1	20 first-year surgical residents with minimal lap. and no robotic exp
Teishima et al. [37], 2012	Lap. → R	10 lap. exp. urologic surgeons (RN)	4 repetitions on da Vinci-Trainer of: Pick and Place Peg Board Rope Walk Energy Dissection Suture Sponge Thread the Rings	13 lap. inexp. urologic surgeons (RN)
Thomaier et al. [38], 2017	Lap. → R	20 medical students (SN) practice on lap. simulator	Baseline evaluation of Peg Transfer on lap. simulator and Peg Board 1 on robotic simulator Participants then undergo practice session on assigned platform Reassessed performing the Peg Transfer task on both simulators	20 medical students (SN) practice on robotic simulator
Tillou et al. [39], 2016	Lap. → R	20 senior residents, registrars and attending surgeons (lap. trained)	To ensure progressive learning, the following task sequence was used on da Vinci Skills simulator: Camera Targeting 1 & 2 Ring Walk 1 & 2 Suture Sponge 1 & 2 Energy Switching 1 and Dissection Participants could only start next task upon scoring ≥ 80% in previous one	8 junior residents (with no formal lap. training) 2 experienced robotic surgeons
Vurgun et al. [40], 2020	Lap. → R	6 medical students (SN) undergo 20 min of lap. training 6 medical students (SN) undergo 40 min of lap. training	Participants undergo assigned lap. training Participants then complete Rope Passing and Ball Placement tasks on Laparo Advance box trainer (Laparo LLC, Wroclaw, Poland) and then similar tasks on Senhance robotic simulator (Senhance Surgical System & Kroton LLC, Warsaw, Poland)	11 medical students (SN) receive no lap. training
Yoo et al. [41], 2015	Lap. → R	11 lap. exp. surgeons (no robotic experience)	1 trial on da Vinci-trainer of: Pick and Place Peg Board Match Board	14 medical students (SN)
Zihni et al. [42], 2016	Lap. → R	26 RN	Tasks performed 3 times each on each platform (Robotic/Lap.). Platform and task order randomised: Peg Transfer Pattern Cutting Intracorporeal Suturing	Comparisons made on platform order

*conv. lap.* conventional laparoscopy, *exp*. experience, *inexp*. inexperienced, *lap*. laparoscopic, *LN* laparoscopic naïve, *R* robotic, *RCT* randomised controlled trial, *RN* robotics naïve, *SN* surgically naïve

### Risk of bias

The results of the risk of bias assessment are depicted in Appendices 1 and 2. Two studies were assessed to have low risk of bias [23, 38] while nineteen studies were judged to have some concerns over risk of bias. Eight studies were deemed to be at high overall risk of bias.

### Findings

The heterogeneity in study design, participants, interventions and outcome measures prevented the undertaking of a meta-analysis so the results were narratively synthesised, categorised into the type of setting and then into either transfer of open skills or transfer of laparoscopic skills.

### Real-time skills transfer

Four studies [14–17] evaluated skill transfer in the clinical setting (Table 3). One study [14] demonstrated the successful transfer of open skills to robotic prostatectomy, describing an experienced open surgeon (with robotic experience of a day’s training course and two cadaveric procedures) achieving a 4-h proficiency after just 12 patients. All outcomes were satisfactory and equivalent to the performances of ‘expert’ surgeons who had experience of over 100 robotic procedures.

**Table 3:** Main results of included studies assessing skill transfer in clinical settings

Study author, year	Relevant outcome measures	Statistical analysis	Key findings	Skill transfer demonstrated?
Ahlering et al. [14], 2003	Operative time, time of completion of each operative step EBL Hb drop Length of hospital stay Resection margin status Continence and potency	Not reported	4-h proficiency achieved after 12 cases Satisfactory outcomes in all metrics	Yes—successful transfer of open surgical skills
Eddib et al. [15], 2013	Total operative time, console time, closing time, procedure time EBL Hb drop Length of hospital stay Pain medication use Complications	Student’s *t* test	Lap. exp. vs lap inexp. surgeons: Mean procedure time: 121 vs 174 min (*p* < 0.05) Mean console time: 70 vs 119 min (*p* < 0.05) Mean EBL: 64 vs 84 ml (*p* = 0.3) Mean Hb drop: 1.7 vs 1.33 (*p* = 0.2) Pain medication use: 74.9 vs 68.8 mg morphine (*p* = 0.83) Length of stay: 1.07 vs 1.35 days (*p* = 0.29)	Advanced lap. experience only significantly impacted length of procedure but no other variables
Kilic et al. [16], 2012	Suture time	*t* test	Lap. exp. surgeons: significant time reduction from laparoscopy to the robot (457 ± 158 s vs 337 ± 235 s, *p* = 0.02) LN participants: non-significant time reduction (*p* = 0.5) No significant differences between lap. exp. and LN mean suturing times on either platform (laparoscopy: *p* = 0.009, robot: *p* = 0.5)	Yes—previous lap. experience improves the robotic surgery learning curve
Kim et al. [17], 2014	Operative time Conversion rate to open surgery Time to first flatus Time to soft diet resumption Length of hospital stay Post-operative morbidity or mortality Complications Resection margin status	Pearson’s Chi-squared test Fisher’s exact test Student’s *t* test Moving average method	Less exp. surgeon had shorter operation time (272.6 ± 121.8 vs 344.0 ± 59.7 min, *p* < 0.001) Lap. exp. surgeon’s patients had shorter hospital stays (8.7 ± 3.7 vs 12.7 ± 12.9 days, *p* = 0.003) and less time to soft diet resumption (3.4 ± 1.4 vs 6.5 ± 5.6 days, *p* < 0.001) Bowel motility: no significant difference in time to first flatus (p = 0.462)	No—substantial previous lap. experience does not translate into superior performances

*EBL* estimated blood loss, *exp*. experienced, *Hb* haemoglobin, *inexp*. inexperienced, *lap*. laparoscopic, *LN* laparoscopic-naïve

Three studies [15–17] assessed the transfer of laparoscopic skills to robotic surgery in the clinical setting. Eddib et al. [15] evaluated robot-assisted laparoscopic hysterectomy performances with the only significant difference reported being that the advanced laparoscopic surgeons had a shorter mean procedure time (121 vs 174 min, *p* < 0.05) with comparable performances in all other metrics. Kilic et al. [16] also reported time reductions for laparoscopically trained participants, concluding that prior laparoscopic experience contributes to an improved robotic surgery learning curve in the context of vaginal cuff closure. In contrast, IK. Kim et al. [17] evaluated the first 100 robot-assisted rectal cancer resections performed by a highly experienced laparoscopic surgeon and a surgeon with limited laparoscopic experience, finding comparable results overall.

### Simulated setting

Table 4 illustrates the findings of the studies assessing skill transfer in the simulated setting. Two studies [22, 30] assessed the transfer of open skills to robotic surgery, with both using the da Vinci Skills Simulator. Cumpanas et al.’s [22] cohort study found no significant difference in the overall performance of open surgeons and surgical novices on the basic robotic task of Peg Board (80 vs 78%, *p* = 0.5) but as task complexity increased, the open surgeons performed significantly better. Conversely Kowalewski et al. [30] found no significant differences in the performances of open surgical novices, intermediates and experts on the Peg Board, Pick and Place and Suture Sponge tasks, with novices outperforming experts on the Thread the Ring task.

**Table 4:** Main results of included studies assessing skill transfer in simulated settings

Study author, year	Relevant outcome measures	Statistical analysis	Key findings	Skill transfer demonstrated?
Anderberg et al. [18], 2010	‘Grab the needle’ time ‘Continuous suturing’ time ‘Tie a knot’ time Damage to skin pad Number of times needle dropped Number of times thread torn	Wilcoxon’s signed rank test for paired samples Mann–Whitney *U* test of two independent samples	Grab the needle: no significant differences Continuous suturing: R. lap. last (conv. lap. first) outperformed R. lap. first group (144 ± 70 vs 216 ± 123 s, *p* = 0.049) Tying a knot: R. lap last (conv. lap first) outperformed R. lap first group (91 ± 35 vs 152 ± 94 s, *p* = 0.004)	Yes—successful lap. skill transfer for advanced task
Angell et al. [19], 2013	Time to complete exercise Errors in technique	Student’s *t* test	Statistically significant improvement in mean time (9.04 vs 16.72 min *p* = 0.0002) and errors (3.57 vs 6.21 errors, *p* = 0.02) for robotic performance before and after laparoscopic training After laparoscopic training, task was completed faster (*p* < 0.001) and with fewer errors (*p* < 0.01) robotically compared to laparoscopically despite no robotic training	Yes—successful lap. skill transfer
Ashley et al. [20], 2019	Time to complete exercise	Two-sample *t* test Paired *t* test Fisher’s exact test or Chi-squared test for categorical data	Degree of improvement was not significantly better than 2 rounds of practice on the practiced modality (*p* = 0.98)	No evidence of lap. skill transfer
Blavier et al. [21], 2007	Performance score Error score Ambidexterity score	Repeated measures analysis of variance Newman–Keuls test for post hoc comparisons	After change to the alternate platform, performance decreased reaching the same score as the 1st trial indicating no skill transfer	No evidence of lap. skill transfer
Cumpanas et al. [22], 2017	Overall percentage score Time to complete exercise Objects dropped Number of times instrument out of view Economy of motion Instrument collisions Misapplied energy time Excessive instrument force Master workspace	Two-tailed paired *t *test	Peg Board: open surgeons achieved better economy of motion (145 vs 167 cm, p = 0.012) and drops (0.18 vs 0.66, p = 0.013) scores with SN achieving better master workspace (9 vs 11, *p* = 0.016) Energy dissection: open surgeons significantly outperformed SN in all metrics except master workspace where they achieved a worse score than SN (16.3 vs 15, *p* = 0.03) Suture Sponge: open surgeons significantly outperformed SN in all metrics except master workspace where they achieved a similar score to SN (8.1 vs 8.4, *p* = 0.3)	Yes—more open surgical skill transfer occurs as task complexity increases
Davila et al. [23], 2017	Performance score (based on time taken and number of errors)	One-way analysis of covariance (ANCOVA)	Intracorporeal Knot Tying scores improved significantly after FLS training compared to dVSS (*p* = 0.018) and no training (*p* = 0.005) Peg Transfer scores improved significantly after FLS training compared to no training (*p* = 0.01)	Yes—successful lap. skill transfer
Feifer et al. [24], 2010	ProMIS performance score LapSim performance score	Mann–Whitney *U* test	Significant performance improvement in all 4 robotic tasks were identified in the dual training group (*p* < 0.05) Participants in the no training group performed worse in all tasks and showed no significant performance improvement	Yes—successful lap. skill transfer
Finnerty et al. [25], 2016	Performance score Economy of motion Time to complete Instrument collisions Master workspace range Critical errors Instruments out of view Excessive instrument force Missed targets Object drops Misapplied energy time	Fisher’s exact test and Chi-squared test Student’s *t *test and Mann–Whitney *U* test Logistic and linear regressions	Matchboard and Energy Dissection median scores did not significantly differ between groups (*p* = 0.27 and 0.99 respectively) Senior surgical residents achieved the highest median Suture Sponge score (*p* = 0.039) Suture Sponge performance significantly correlated with number of lap. cases logged during residency (*p* = 0.005, *r*^2^ = 0.21)	Yes—increased lap. skill transfer seen for advanced task
Hagen et al. [26], 2009	Time to complete exercise	t-test	Lap. exp. participants achieved a better robotic task performance than lap. novices (*p* < 0.05)	Yes—lap. exp. correlates with robotic skill
Hassan et al. [27], 2015	Time to complete exercise Object drops Instrument collisions Number of times instrument out of view Excessive instrument force	Mann–Whitney *U* test of two independent samples	Pick and Place: mean time on robotic platform was 41 s without previous lap. exp. vs 35 s with lap. exp. (*p* = 0.2) Thread the Ring: no significant difference in mean time on robotic platform for no previous lap. exp. vs those with exp. (212 vs 216 s (*p* = 0.66))	No evidence of lap. skill transfer
Jayaraman et al. [28], 2010	Procedure time Complications Number of bites for front and back wall Anastomotic integrity	Student’s *t* test Chi-squared test	Lap exp. surgeon rapidly overcame his learning curve for the R. procedure (36.8 ± 5.8 vs. 24.7 ± 0.6 min, *p* = 0.02) and achieved plateau after 3 cases Increased minimally invasive surgical exp. correlated with decreased probability of anastomotic leak	Yes—lap. exp. contributed to rapid achievement of proficiency and better anastomosis
Kim et al. [29], 2014	Time to complete each exercise	Mean values calculated for continuous data Fisher’s exact test or Chi-squared test for categorical data	Robotic tasks were performed better and faster by lap. exp. participants compared to lap. novices but only ‘suturing and knot tying’ task achieved statistical significance (*p* = 0.011 in 1st trial, *p* = 0.003 in 2nd trial)	Yes—successful lap. skill transfer for advanced task
Kowalewski et al. [30], 2018	Overall performance score Time to complete each exercise Economy of motion Instrument collisions Excessive instrument force Instruments out of view Master workspace range Object drops Missed targets	Analysis of variance (ANOVA) Kruskal–Wallis test for non-parametric data	Thread the Ring task: both open and lap. novices outperformed experts (*p* = 0.002 and *p* = 0.004, respectively) No significant differences between groups for the Peg Board, Pick and Place and Suture Sponge tasks	No evidence of either open or lap. skill transfer
McVey et al. [31], 2016	Time to complete each exercise Global rating score using Likert scale provided by two blinded experts	Paired sample *t* test Wilcoxon matched-pairs test ANOVA	Baseline lap. task time of trainees on Intracorporeal Suturing with Knot Tying (ISKT) task correlated with post-robotic performances of Peg Transfer (*r* = 0.480) and ISKT time (*r* = 0.529, *p* < 0.01)	Yes—successful lap. skill transfer for advanced task
Moncayo et al. [32], 2019	Time to complete each exercise Objective Structured Assessment of Technical Skills (OSATS)	Paired *t *test Wilcoxon test Mann–Whitney *U* test	Thread the Ring task: transfer of laparoscopic skills to robotic skills was statistically significant (*p* = 0.01) Transfer the Plot task: no statistically significant transfer effect was seen (*p* = 0.96)	Yes—successful lap. skill transfer for advanced task
Obek et al. [33], 2005	Overall performance score	Paired *t* test for within-group comparison *t* test	Post-training scores increased significantly from 57.4 to 81.8 for the lap. trained group (*p* = 0.02) and from 27.4 to 66.1 for the R. trained group (*p* = 0.04) Statistically significant decrease in error score in the lap. trained group, from 42.1 to 16.2 (*p* = 0.02)	Yes—evidence of incomplete lap. skill transfer
Panait et al. [34], 2014	Overall performance score	Paired *t* test	Lap. exp. trainees achieved similar robotic and laparoscopic scores for Circle Cutting (64 ± 9 vs 69 ± 15, *p* > 0.05) and Intracorporeal Suturing tasks (95 ± 3 vs 92 ± 10, *p* > 0.05)	Yes—lap. skill transfer demonstrated for more advanced tasks
Passerotti et al. [35], 2015	Time to complete each exercise	Statistical models created using generalised estimating equations with Poisson’s marginal distribution and link logarithmic function Interactions between the variables were assessed using Bonferroni multiple or post hoc comparison	Peg Transfer: lap. exp. and novice participants both achieved learning curve plateau after 3^rd^ session although exp. participants had a lower average completion time than novices at every corresponding session (*p* < 0.01) Precision cutting: lap. exp. participants achieved learning curve plateau after 2nd session, novices after 4th session. Exp. participants had a lower average completion time than novices at every corresponding session (*p* < 0.01) Simple suturing with intracorporeal knot tying: lap. exp. participants showed no improvement from the first to last sessions (*p* = 0.50) but completed the task faster than novice participants at every corresponding session	Yes – lap. skill transfer demonstrated, most evident in the advanced tasks
Pimentel et al. [36], 2018	Overall performance score Time to complete exercise Object drops Economy of motion Excessive instrument force Instrument collisions Instruments out of view Master workspace range Missed targets	Mann–Whitney *U* test Friedman test	No statistically significant difference in overall score between lap. exp. and LN for all tasks: Peg Board 2 (*p* = 0.57), Ring and Rail 1 (*p* = 0.113), Suture Sponge 1 (*p* = 0.67)	No evidence of lap. skill transfer
Teishima et al. [37], 2012	Overall performance score Time to complete exercise Critical errors Number of drops Economy of motion Excessive instrument force Instrument collisions Master workspace range	Mann–Whitney *U* test Wilcoxon test	No significant differences between groups for Pick and Place, Peg Board, Rope Walk, Thread the Rings and Energy Dissection tasks Participants with more lap. exp. performed significantly better in the 2nd (*p* = 0.0236) and 3rd trials (*p* = 0.0043) of Suture Sponge than less exp. counterparts but no significant difference in 4th trial (*p* = 0.1068)	Yes—limited evidence of lap. skill transfer
Thomaier et al. [38], 2017	Time to complete exercise Error rate Mimic da Vinci-trainer motion metrics Modified global rating score	Student’s *t* test	Decrease in mean completion time for lap. trained group (120 vs 167 s, *p* = 0.004) R. trained group had significantly faster (*p* < 0.001) and better robotic task performance with higher global rating scale scores (*p* = 0.006) compared to the lap. trained group	Yes—successful but incomplete lap. skill transfer
Tillou et al. [39], 2016	Performance index Time to complete exercise Instrument collisions Economy of movement Excessive instrument force Instruments out of view Workspace size	Dunn’s multiple comparisons test	No significant differences between junior and senior resident scores and the scores of attending surgeons (*p* = 0.45) Attending surgeons needed more exercise repetitions than registrars (*p* = 0.024)	No evidence of lap. skill transfer
Vurgun et al. [40], 2020	Time to complete exercise Clutch use Errors (number of ball drops, instruments out of view, interruptions requiring manual adjustments)	Pearson Chi-squared test Independent-samples Kruskal–Wallis test	Ball placement task: no significant differences between groups for any metric Rope Passing task: no significant differences between groups for any metric	No evidence of lap. skill transfer
Yoo et al. [41], 2015	Overall performance score Time to complete exercise Number of drops Economy of motion Instruments out of view Excessive instrument force Instrument collisions Master workspace range	Independent-sample *t* test Mann–Whitney *U* test	No significant differences between overall scores of lap exp. participants and SN on Pick and Place (67.55 vs 75.71%, *p* = 0.337), Peg Board (51.36 vs 41.56%, *p* = 0.239) and Match Board (43.30 vs 42.31%, *p* = 0.706) tasks Lap exp. participants scored significantly better in the number of instrument collisions (4.11 ± 2.98 vs. 1.18 ± 1.60, *p* = 0.016), and the number of drops (2.67 ± 1.80 vs. 1.00 ± 1.01, p = 0.028),	No significant evidence of lap. skill transfer
Zihni et al. [42], 2016	Time to complete exercise Errors per trial (EPT)	Unpaired Student’s *t* test	Lap. trained group made more errors on the robotic Pattern Cutting task (1.86 vs 1.03 EPT, *p* = 0.02) No significant differences between the groups for Peg Transfer or Intracorporeal Suturing	No evidence of lap. skill transfer

*conv. lap.* conventional laparoscopy, *dVSS* da Vinci Surgical System, *exp*. experience, *FLS* Fundamentals of Laparoscopic Surgery, *lap*. laparoscopic, *LN* laparoscopic naïve, *R* robotic, *RN* robotics naïve, *SN* surgically naïve

Twenty-four studies [18–21, 23–42] evaluated the transfer of laparoscopic skills to robotic surgery in the simulated setting. Thomaier et al. [38] assessed the performance of a basic robotic task (Peg Transfer) by surgical novices trained on either a laparoscopic or a robotic simulator, reporting that the laparoscopic trained group successfully transferred their skills to the robot with the mean time to complete the robotic task decreasing from baseline (167 vs 120 s, *p* = 0.004) with significant improvements in the global rating composite score and instrument collisions score. The transfer is incomplete, however, as the laparoscopic trained group were outperformed on the robotic platform by the robot trained group.

Three studies [24, 26, 35] reported successful skill transfer in both basic and advanced tasks. Hagen et al. [26] found that laparoscopically trained surgeons significantly outperformed laparoscopically naïve participants on ring placement, suturing with knot tying and needle guidance tasks, concluding that laparoscopic experience is a strong predictor of robotic performance. Feifer et al. [24] described significant performance improvements in all robotic tasks (Peg Transfer, Cutting, Intracorporeal Knot Tying and Cannulation) for surgical novices who had received dual training on augmented reality (ProMIS) and virtual reality (LapSim) laparoscopic simulators, with no significant improvements observed in those who had received no training and fewer significant improvements in participants who had trained on either simulator alone.

Ten studies [18, 19, 23, 25, 28, 29, 31–34] found that laparoscopic skill transfer was most evident when advanced surgical tasks such as intracorporeal knot tying or suturing were performed. In an RCT conducted by Davila et al. [23], the mean score improvement for robotic intracorporeal knot tying was greatest for inexperienced surgical trainees who had undergone 4 weeks of laparoscopic training compared to those who instead received 4 weeks of robotic training (82.2 vs 40.5 points, *p* = 0.018). In accordance with the findings of Davila et al., Panait et al.’s [34] cohort study reported that performances on the robot equalled those on the laparoscopic platform as the complexity of tasks increased thereby demonstrating transfer of skills.

Finnerty et al. [25] echoed these findings, reporting a statistically significant correlation between the number of laparoscopic operations logged by surgical trainees and performance on an advanced robotic task only (Suture Sponge) (*p* = 0.005, *r*^2^ = 0.21). Jayaraman et al.’s [28] study also reported that increased minimally invasive surgical experience correlated with a decreased likelihood of anastomotic leak when performing the complex task of suturing a biliary-enteric anastomosis in robotic choledochojejunostomies on a porcine model.

McVey et al.’s [31] cohort study found that baseline laparoscopic task time of surgical trainees on the advanced Intracorporeal Suturing with Knot Tying (ISKT) task, but not the basic Peg Transfer (PT) task, correlated with post-robotic training performances of both Peg Transfer and ISKT time. In a similar fashion, Moncayo et al. [32] described significant skill transfer for laparoscopically trained students to the confined space of the simulated paediatric robotic platform for the advanced Thread the Ring task (*p* = 0.01) but not for the basic Transfer the Plot task (*p* = 0.96). Obek et al. [33] evaluated the performances of laparoscopically trained students on the advanced knot tying robotic task, reporting that their composite score significantly increased after training (43% improvement, *p* = 0.02) with a significant decrease in error score (16.2 vs 42.1, *p* = 0.02) thereby demonstrating skill transfer to the robot.

In contrast, nine studies [20, 21, 27, 30, 36, 39–42] found no evidence of skill transfer in either basic or advanced tasks. Tillou et al.’s [39] study involved assessment of the more advanced task of suturing alongside basic tasks such as camera manipulation and Endowrist handling, finding comparable performances across all tasks between laparoscopically experienced surgeons and laparoscopically naïve trainees thereby signifying a complete lack of skill transfer. Similarly, Zihni et al. [42] found no evidence of laparoscopic skill transfer in both basic and advanced robotic tasks, noting that prior performance of the Pattern Cutting task on the laparoscopic platform appeared to impede subsequent performance of the task on the robot. Teishima et al. [37] reported significantly better overall Suture Sponge scores in the initial trials for laparoscopic experts compared to surgeons with less experience but by the fourth trial, there was no significant difference in scores (59.1 ± 19.5 vs 49.1 ± 7.0, *p* = 0.1068) suggesting that laparoscopic experience only confers an advantage in the initial phase of the robotic learning curve.

## Discussion

Robotic training curricula consist of multiple modalities aiming to develop the necessary cognitive and psychomotor skills for safe robotic surgical practice. These modalities include dry and wet laboratory training, virtual reality simulation and online lectures [43]. Skill transfer from robotic virtual reality simulation to the operative environment has been demonstrated, thereby indicating its integral role in training curricula [44]. This review aimed to evaluate the presence of crossover from a trainee’s open or laparoscopic skills as any such skill transfer might accelerate the robotic training process and also result in greater availability of expensive robotic simulators to other trainees as less time is spent in the simulation phase. The results of the 29 included studies found conflicting evidence relating to the transfer of open and laparoscopic skills to robotic surgery in both clinical and simulated settings.

Successful open surgical skill transfer to the robotic clinical setting was demonstrated with satisfactory peri- and post-operative outcomes achieved in the absence of any laparoscopic experience [14]. However, the findings of the studies conducted in the simulated setting confirm the current view in surgical practice that open surgical skill transfer of any extent is insufficient in itself to enable a direct transition to robotic surgical practice in the absence of any systematic robotic training; one study found no evidence of any skill transfer [30] while the other reported skill transfer only for advanced robotic tasks [22].

Laparoscopic surgical experience only translates into reduced robotic procedure time in the clinical setting, with no significant improvement in other metrics [15, 16]. One study [17] contrarily reported that the surgeon with the least laparoscopic experience had a significantly shorter mean operative time but acknowledged confounders such as differing specimen extraction and anastomosis techniques between the surgeons which might have affected procedure time. Successful laparoscopic skill transfer does occur in the simulated setting, most notably when advanced robotic tasks are performed [37] although some studies did observe a transfer effect for basic tasks as well [23, 24, 26, 35, 38]. Substantial laparoscopic experience is not essential for successful skill transfer to occur as six studies [18, 19, 24, 32, 33, 38] reported a transfer effect in surgical novices following their completion of short laparoscopic training courses.

Thus, for both open and laparoscopic modalities, several studies suggested a possible correlation between task complexity and the extent of skill transfer. This is consistent with the high construct validity (ability to differentiate between experts and novices) associated with advanced robotic tasks such as Suture Sponge [45], so any superiority in the performances of open or laparoscopically experienced participants would be most evident in these tasks. Cumpanas et al. [22] attributed open surgeons’ extensive prior experience in guiding the needle from different angles when performing suturing as the reason for the transfer effect seen but noted that this inclination to reproduce their usual open surgery hand movements on the robotic console resulted in a worse master workspace score (a metric representing the virtual space used by the instruments during task performance). In contrast, Pimentel et al. [36] reported significantly better master workspace scores for laparoscopic surgeons as they are accustomed to manoeuvring instruments in a confined space which suggests that, unlike open surgical experience, prior laparoscopic experience enhances efficiency of movement on the robotic platform.

As described for open surgery [22], another reason for the laparoscopic transfer effect observed on advanced tasks may include an existing skillset of needle driving developed through repetitive practice and experience which predisposes laparoscopically trained participants to a more precise performance on the robot [25, 26, 29]. Two studies [24, 33] also conjectured that laparoscopic training facilitates the development of visual cues associating knot tension with suture resistance, thus preparing the participant for the loss of haptic feedback on the robotic platform but this is contrary to existing literature which reported that the perception of haptic feedback on the robot is similar between novices and laparoscopic surgeons [46].

There may be a preliminary period in which laparoscopically trained subjects transitioning to robotics adopt a conservatory strategy with camera and instrument movements [21] as they ‘unlearn’ certain laparoscopic-associated acquisitions [39] such as adjustment for the fulcrum effect [41] to adapt to the robotic console. This could explain why, for studies in which a progressively difficult task sequence was employed, performances improved as task complexity increased because the initial basic robotic tasks may serve to prime and enable modification of the laparoscopic skillset to the robot. This, therefore, implicates a role for systematic robotic training to include both basic and advanced tasks regardless of a surgeon’s prior laparoscopic experience.

### Limitations

Limitations to the review findings include the low quality of certain studies, small sample sizes and low number of task repetitions assessed in some of the included studies. Reporting of outcomes also varied between studies, thus preventing a meta-analysis of results, with some studies electing to use time and score measurements only which precludes comprehensive assessment of surgical performance [47]. Confounders for laparoscopic skill transfer studies such as open surgical experience [22, 29] and video gaming experience [48] were inconsistently reported with variation in the definition of ‘laparoscopically experienced’; having more than 3 previous laparoscopic experiences constituted ‘experienced’ in one study [16] whereas participants with up to 750 laparoscopic cases logged were classed as ‘experienced’ in another [25]. At review level, the exclusion of non-English language studies may have increased the risk of language bias. However, strengths of the review include its comprehensive search strategy of numerous databases, adherence to the PRISMA [9] and SWiM [13] checklists, thorough quality assessment and its nature as the first systematic review investigating skill transfer of these modalities.

### Implications for research and/or practice

All studies in the simulated setting assessed only the initial phase of the robotic surgery learning curve where skill transfer is most evident [37], so increasing the numbers of trials up to the acquisition of proficiency [23] in future studies would enable evaluation of skill transfer in the context of the whole learning curve. Given the reported additive mechanism by which a combination of virtual reality and augmented reality simulators enhance robotic performances [24], high-quality randomised controlled trials evaluating their impact on the robotic surgery learning curve are warranted especially as they can be adapted for use with robotic training modules [24] which may enable development of more cost-effective robotic surgery training curricula. Some studies also evaluated a robotic transfer effect to laparoscopy [20, 27, 32, 33, 38, 42] with contradictory findings, so further investigation of the impact of concomitantly teaching open, laparoscopic and robotic skills on trainee performances across the various modalities may aid in the development of more effective curricula to accelerate skill acquisition. Cohort studies with larger groups of surgeons in the intraoperative setting will enable more confident conclusions to be drawn regarding open and laparoscopic skill transfer in the clinical setting.

Regarding clinical practice, although all clinical studies [14–17] concluded that robotic skills can be acquired in the absence of any laparoscopic experience, open and laparoscopic training remains an essential part of robust surgical training. Robotic surgeons still employ laparoscopic skills such as pneumoperitoneum creation and adhesiolysis to facilitate port insertion [49], and they must be well-versed in open surgical techniques given the rates of conversion from robotic to open being as high as 9.2% for nephroureterectomy, for example [50]. Undergoing concomitant training across all modalities enables trainees to become more clinically skilled surgeons, able to safely and effectively undertake a variety of procedures thereby increasing the range of care they can provide. With results suggesting that laparoscopic skill transfer occurs in the initial phase of the learning curve, there is need for individually tailored curricula correlating with the level of laparoscopic expertise the trainee has.

## Conclusion

Skill transfer from both modalities appears to be most apparent when advanced robotic tasks are performed in the initial phase of the learning curve but quality and methodological limitations of the existing literature prevent definite conclusions from being drawn. The impact of incorporating laparoscopic simulation into robotic training curricula on all phases of the robotic surgery learning curve and on the cost effectiveness of training should be investigated.

## Supplementary Information

Below is the link to the electronic supplementary material.

Supplementary file1 (DOCX 1284 KB)

## References

[1] Lam K, Clarke J, Purkayastha S, Kinross JM (2021). Uptake and accessibility of surgical robotics in England. Int J Med Rob Comput Assist Surg.

[2] Lanfranco AR, Castellanos AE, Desai JP, Meyers WC (2004). Robotic surgery: a current perspective. Ann Surg.

[3] Cao L, Yang Z, Qi L, Chen M (2019). Robot-assisted and laparoscopic vs open radical prostatectomy in clinically localized prostate cancer: perioperative, functional, and oncological outcomes: a Systematic review and meta-analysis. Medicine (Baltimore).

[4] Skupin PA, Stoffel JT, Malaeb BS, Barboglio-Romo P, Ambani SN (2020). Robotic versus open ureteroneocystostomy: is there a robotic benefit?. J Endourol.

[5] Brook N, Dell’Oglio P, Barod R, Collins J, Mottrie A (2019). Comprehensive training in robotic surgery. Curr Opin Urol.

[6] Moglia A, Ferrari V, Morelli L, Ferrari M, Mosca F, Cuschieri A (2016). A systematic review of virtual reality simulators for robot-assisted surgery. Eur Urol.

[7] Li MM, George J (2017). A systematic review of low-cost laparoscopic simulators. Surg Endosc.

[8] Hertz AM, George EI, Vaccaro CM, Brand TC (2018). Head-to-head comparison of three virtual-reality robotic surgery simulators. JSLS J Soc Laparoendosc Surg.

[9] Moher D, Liberati A, Tetzlaff J, Altman DG (2009). Preferred reporting items for systematic reviews and meta-analyses: the PRISMA statement. BMJ.

[10] Chahal B, Aydin A. The transfer of open and laparoscopic surgical skills to robotic surgery: a systematic review PROSPERO 2021 CRD42021231235. Available from: https://www.crd.york.ac.uk/prospero/display_record.php?ID=CRD42021231235, Accessed 15 Mar 2021

[11] Sterne JAC, Savović J, Page MJ, Elbers RG, Blencowe NS, Boutron I, Cates CJ, Cheng H-Y, Corbett MS, Eldridge SM, Hernán MA, Hopewell S, Hróbjartsson A, Junqueira DR, Jüni P, Kirkham JJ, Lasserson T, Li T, McAleenan A, Reeves BC, Shepperd S, Shrier I, Stewart LA, Tilling K, White IR, Whiting PF, Higgins JPT (2019). RoB 2: a revised tool for assessing risk of bias in randomised trials. BMJ.

[12] Sterne JAC, Hernán MA, Reeves BC, Savović J, Berkman ND, Viswanathan M, Henry D, Altman DG, Ansari MT, Boutron I, Carpenter JR, Chan AW, Churchill R, Deeks JJ, Hróbjartsson A, Kirkham J, Jüni P, Loke YK, Pigott TD, Ramsay CR, Regidor D, Rothstein HR, Sandhu L, Santaguida PL, Schünemann HJ, Shea B, Shrier I, Tugwell P, Turner L, Valentine JC, Waddington H, Waters E, Wells GA, Whiting PF, Higgins JPT (2016). ROBINS-I: a tool for assessing risk of bias in non-randomized studies of interventions. BMJ.

[13] Campbell M, McKenzie JE, Sowden A, Katikireddi SV, Brennan SE, Ellis S (2020). Synthesis without meta-analysis (SWiM) in systematic reviews: reporting guideline. BMJ.

[14] Ahlering TE, Skarecky D, Lee D, Clayman RV (2003). Successful transfer of open surgical skills to a laparoscopic environment using a robotic interface: initial experience with laparoscopic radical prostatectomy. J Urol.

[15] Eddib A, Jain N, Aalto M, Hughes S, Eswar A, Erk M (2013). An analysis of the impact of previous laparoscopic hysterectomy experience on the learning curve for robotic hysterectomy. J Robot Surg.

[16] Kilic GS, Walsh TM, Borahay M, Zeybek B, Wen M, Breitkopf D (2012). Effect of residents' previous laparoscopic surgery experience on initial robotic suturing experience. ISRN Obstet Gynecol.

[17] Kim IK, Kang J, Park YA, Kim NK, Sohn SK, Lee KY (2014). Is prior laparoscopy experience required for adaptation to robotic rectal surgery? Feasibility of one-step transition from open to robotic surgery. Int J Colorectal Dis.

[18] Anderberg M, Larsson J, Kockum CC, Arnbjörnsson E (2010). Robotics versus laparoscopy—an experimental study of the transfer effect in maiden users. Ann Surg Innov Res.

[19] Angell J, Gomez MS, Baig MM, Abaza R (2013). Contribution of laparoscopic training to robotic proficiency. J Endourol.

[20] Ashley CW, Donaldson K, Evans KM, Nielsen B, Everett EN (2019). Surgical cross-training with surgery naive learners: implications for resident training. J Surg Educ.

[21] Blavier A, Gaudissart Q, Cadiere GB, Nyssen AS (2007). Comparison of learning curves and skill transfer between classical and robotic laparoscopy according to the viewing conditions: implications for training. Am J Surg.

[22] Cumpanas AA, Bardan R, Ferician OC, Latcu SC, Duta C, Lazar FO (2017). Does previous open surgical experience have any influence on robotic surgery simulation exercises?. Wideochirurgia I Inne Techniki Maloinwazyjne.

[23] Davila DG, Helm MC, Frelich MJ, Gould JC, Goldblatt MI (2017). Robotic skills can be aided by laparoscopic training. Surg Endosc Other Intervent Techn..

[24] Feifer A, Al-Almari A, Kovacs E, Delisle J, Carrier S, Anidjar M (2010). Randomized controlled trial of virtual reality and hybrid simulation for robotic surgical training. J Urol.

[25] Finnerty BM, Afaneh C, Aronova A, Fahey TJ, Zarnegar R (2016). General surgery training and robotics: are residents improving their skills?. Surg Endosc.

[26] Hagen ME, Wagner OJ, Inan I, Morel P (2009). Impact of IQ, computer-gaming skills, general dexterity, and laparoscopic experience on performance with the da Vinci® surgical system. Int J Med Rob Comput Assist Surg.

[27] Hassan SO, Dudhia J, Syed LH, Patel K, Farshidpour M, Cunningham SC (2015). Conventional laparoscopic vs robotic training: which is better for Naive users? A randomized prospective crossover study. J Surg Educ.

[28] Jayaraman S, Quan D, Al-Ghamdi I, El-Deen F, Schlachta CM (2010). Does robotic assistance improve efficiency in performing complex minimally invasive surgical procedures?. Surg Endosc.

[29] Kim HJ, Choi G-S, Park JS, Park SY (2014). Comparison of surgical skills in laparoscopic and robotic tasks between experienced surgeons and novices in laparoscopic surgery: an experimental study. Ann Coloproctol.

[30] Kowalewski KF, Schmidt MW, Proctor T, Pohl M, Wennberg E, Karadza E (2018). Skills in minimally invasive and open surgery show limited transferability to robotic surgery: results from a prospective study. Surg Endosc.

[31] McVey R, Goldenberg MG, Bernardini MQ, Yasufuku K, Quereshy FA, Finelli A (2016). Baseline laparoscopic skill may predict baseline robotic skill and early robotic surgery learning curve. J Endourol.

[32] Moncayo S, Compagnon R, Caire F, Grosos C, Bahans C, Ilhero P (2019). Transition effects from laparocscopic to robotic surgery skills in small cavities. J Rob Surg..

[33] Obek C, Hubka M, Porter M, Chang L, Porter JR (2005). Robotic versus conventional laparoscopic skill acquisition: implications for training. J Endourol Endourol Soc.

[34] Panait L, Shetty S, Shewokis PA, Sanchez JA (2014). Do laparoscopic skills transfer to robotic surgery?. J Surg Res.

[35] Passerotti CC, Franco F, Bissoli JCC, Tiseo B, Oliveira CM, Buchalla CAO (2015). Comparison of the learning curves and frustration level in performing laparoscopic and robotic training skills by experts and novices. Int Urol Nephrol.

[36] Pimentel M, Cabral RD, Costa MM, Neto BS, Cavazzola LT (2018). Does previous laparoscopic experience influence basic robotic surgical skills?. J Surg Educ.

[37] Teishima J, Hattori M, Inoue S, Ikeda K, Hieda K, Miyamoto K (2012). Impact of laparoscopic experience on the proficiency gain of urologic surgeons in robot-assisted surgery. J Endourol.

[38] Thomaier L, Orlando M, Abernethy M, Paka C, Chen CCG (2017). Laparoscopic and robotic skills are transferable in a simulation setting: a randomized controlled trial. Surg Endosc.

[39] Tillou X, Collon S, Martin-Francois S, Doerfler A (2016). Robotic surgery simulator: elements to build a training program. J Surg Educ.

[40] Vurgun N, Vongsurbchart T, Myszka A, Richter P, Rogula T (2020). Medical student experience with robot-assisted surgery after limited laparoscopy exposure. J Robot Surg.

[41] Yoo B, Kim J, Cho J, Shin J, Lee D, Kwak J (2015). Impact of laparoscopic experience on virtual robotic simulator dexterity. J Minim Access Surg.

[42] Zihni A, Ge T, Ray S, Wang R, Liang Z, Cavallo JA (2016). Transfer and priming of surgical skills across minimally invasive surgical platforms. J Surg Res.

[43] Azadi S, Green IC, Arnold A, Truong M, Potts J, Martino MA (2021). Robotic surgery: the impact of simulation and other innovative platforms on performance and training. J Minim Invasive Gynecol.

[44] Schmidt MW, Köppinger KF, Fan C, Kowalewski KF, Schmidt LP, Vey J, Proctor T, Probst P, Bintintan VV, Müller-Stich BP, Nickel F (2021). Virtual reality simulation in robot-assisted surgery: meta-analysis of skill transfer and predictability of skill. BJS Open.

[45] Lyons C, Goldfarb D, Jones SL, Badhiwala N, Miles B, Link R (2013). Which skills really matter? Proving face, content, and construct validity for a commercial robotic simulator. Surg Endosc.

[46] Hagen ME, Meehan JJ, Inan I, Morel P (2008). Visual clues act as a substitute for haptic feedback in robotic surgery. Surg Endosc.

[47] Hernandez JD, Bann SD, Munz Y, Moorthy K, Datta V, Martin S (2004). Qualitative and quantitative analysis of the learning curve of a simulated surgical task on the da Vinci system. Surg Endosc.

[48] Hvolbek AP, Nilsson PM, Sanguedolce F, Lund L (2019). A prospective study of the effect of video games on robotic surgery skills using the high-fidelity virtual reality RobotiX simulator. Adv Med Educ Pract.

[49] Sridhar AN, Briggs TP, Kelly JD, Nathan S (2017). Training in robotic surgery-an overview. Curr Urol Rep.

[50] Khanna A, Campbell SC, Murthy PB, Ericson KJ, Nyame YA, Abouassaly R (2020). Unplanned conversion from minimally invasive to open kidney surgery: the impact of robotics. J Endourol.

