# Supplementary material for: Transfer of open and laparoscopic skills to robotic surgery: a systematic review
Source: J Robot Surg. 2022 Nov 22;17(4):1207–25. doi: 10.1007/s11701-022-01492-9 (PMC10374669; doi:10.1007/s11701-022-01492-9)
Supplement: Supplementary file 1 — Supplementary file1 (DOCX 1284 KB) [file 11701_2022_1492_MOESM1_ESM.docx]

# Appendix 1


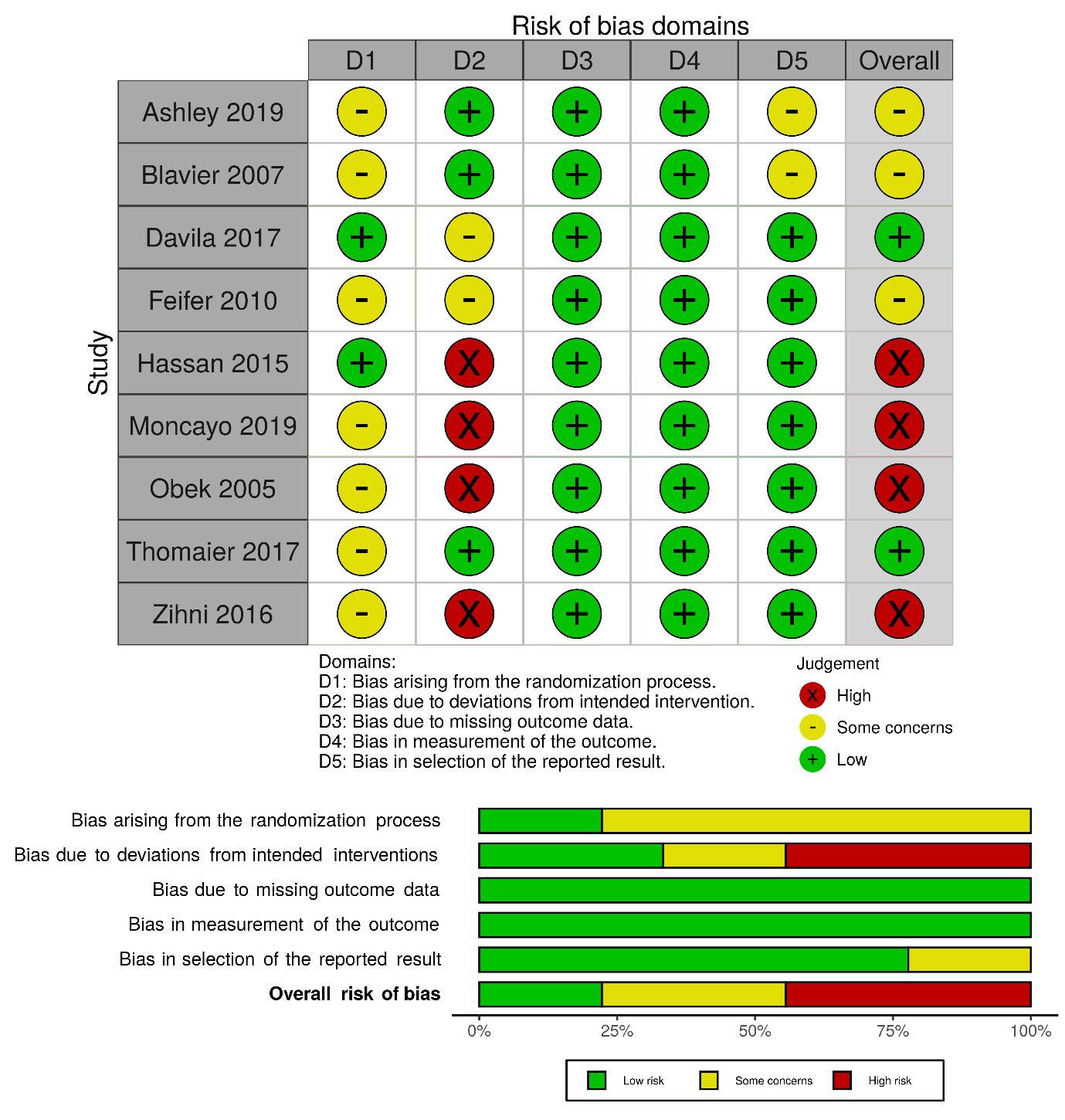
Risk of bias in the RCTs, created using the RobVIS tool

(McGuinness LA, Higgins JPT (2020) Risk-of-bias VISualization (robvis): an R package and Shiny web app for visualizing risk-of-bias assessments. Res Syn Methods. 12:1–7)

# Appendix 2


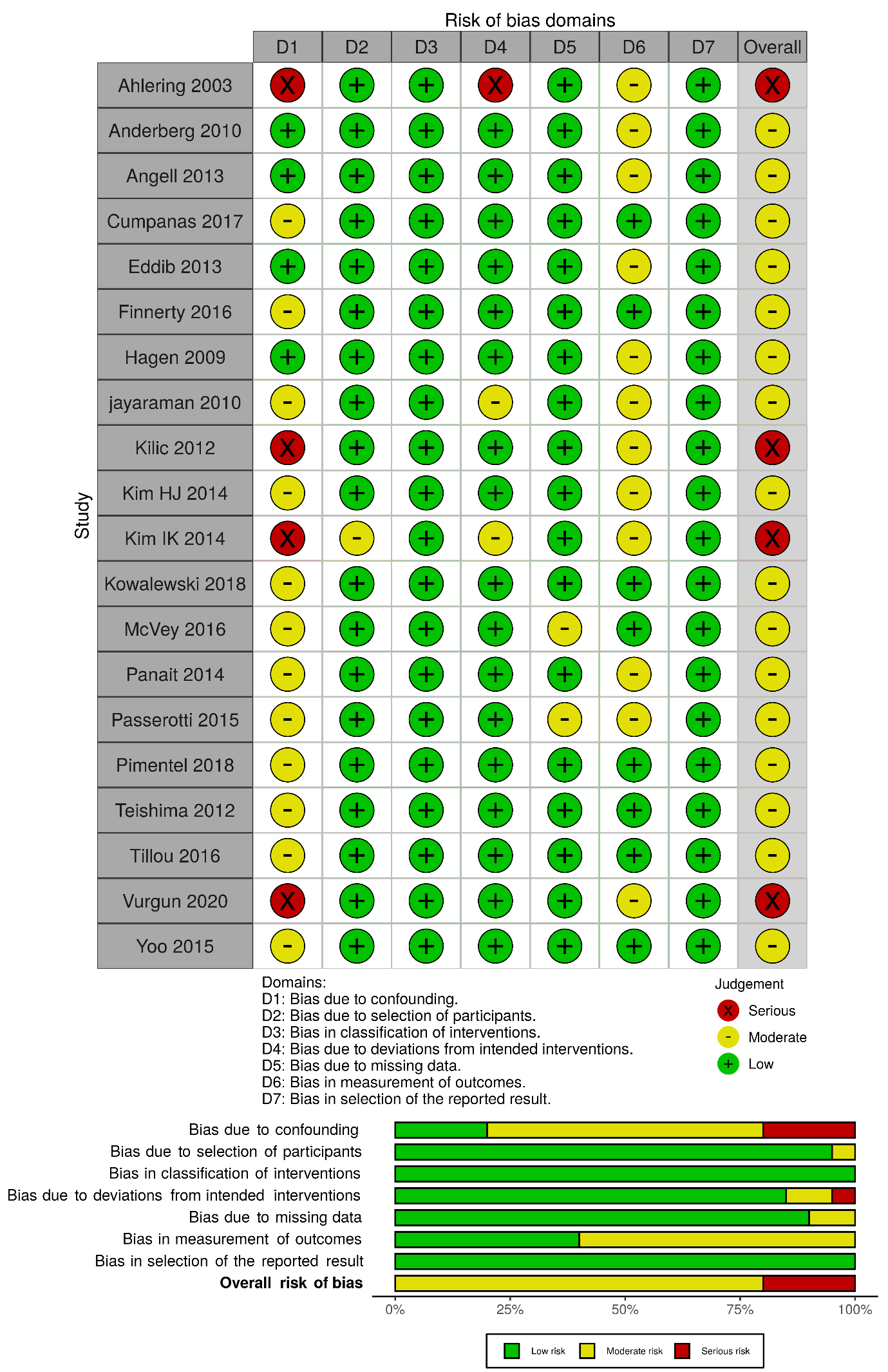
Risk of bias in the non-RCTs, created using the RobVIS tool
